# Aluminum phosphide poisoning with Brugada ECG: a case report highlighting diagnostic challenges arising from patient nondisclosure

**DOI:** 10.1186/s12245-025-00899-z

**Published:** 2025-05-12

**Authors:** Mohammed Ahmed Sadeq, Magdy Soliman Dawood, Reem Mohamed Farouk Ghorab, Marwa Salah Roshdy Aggour

**Affiliations:** 1 Emergency Medicine Department, Elsheikh Zayed Specialized Hospital, Elsheikh Zayed City, Egypt; 2 Misr University for Science and Technology, 6th of October, Egypt

**Keywords:** Aluminum phosphide, Poisoning, Cardiotoxicity, Delayed diagnosis, ECG changes, Cardiac arrest, Supportive care

## Abstract

**Background:**

Aluminum phosphide (AlP) poisoning is a major cause of mortality, often presenting with non-specific symptoms that complicate diagnosis.

**Case:**

A 19-year-old male presented with vomiting, abdominal pain, hypotension, and ECG abnormalities. He initially denied ingestion, delaying treatment. Despite supportive care, he progressed to cardiac arrest and death.

**Conclusion:**

This case illustrates the diagnostic challenges of AlP poisoning and underscores the need for early suspicion and intervention to improve patient outcomes.

**Supplementary Information:**

The online version contains supplementary material available at 10.1186/s12245-025-00899-z.

## Introduction

Aluminum phosphide (AlP) poisoning is a common cause of poisoning-related mortality in many parts of the world, particularly in agricultural regions where the compound is readily accessible, like Egypt [1]. The initial presentation of patients with AlP poisoning includes nausea, vomiting, and abdominal pain. These nonspecific symptoms, coupled with the rapid course of the disease, make management a difficult challenge.

Treatment of AlP poisoning management is conservative, starting with gastric lavage with potassium permanganate and paraffin oil, and symptomatic management with vasopressors, for example. However, the progression of the disease is rapid, and the outcome is seldom positive, with mortality rates ranging from 37 to 100% [2] and death usually occurs within 24 h after exposure [3]. Cardiovascular mortality, whether arrhythmias, myocarditis, or heart failure, remains one of the most common causes of death among AlP poisoning patients [2, 4–8].

This prognostically sinister medical entity can be further complicated by patient non-disclosure, leading to a wider array of differential diagnoses and affecting critical time-sensitive management. A survey published by Agaku et al. [9] showed that 12.3% of respondents withheld information from clinicians out of concern for privacy. Levy et al. [10] have reached a similar conclusion, showing that many respondents intentionally withheld information pertaining to their medical diagnosis, especially when they disagreed with or were misunderstood.

We present a challenging case of AlP poisoning, which was further complicated by the patient denying taking any toxic substance and the slow/insidious progression of the disease.

### Case report

A 19-year-old male patient presented to our emergency department with more than five episodes of vomiting associated with epigastric pain and generalized weakness. The patient had no past medical history. When presented, the patient had a patent secure airway, 99% oxygen saturation on room air, 23 breaths per minute respiratory rate, a blood pressure of 70/40, a heart rate of 110 beats per minute, a Glasgow coma scale of 15, and a random blood sugar of 210 mg/dL. On examination, the patient had bilaterally regular and reactive pupils, normal tongue and mucous membranes, and a lax abdomen with moderate epigastric and right hypochondriac tenderness. On auscultation, the patient’s chest was clear bilaterally. The patient and his family were extensively questioned about toxic materials, which they fervently denied.

**Fig. 1:**
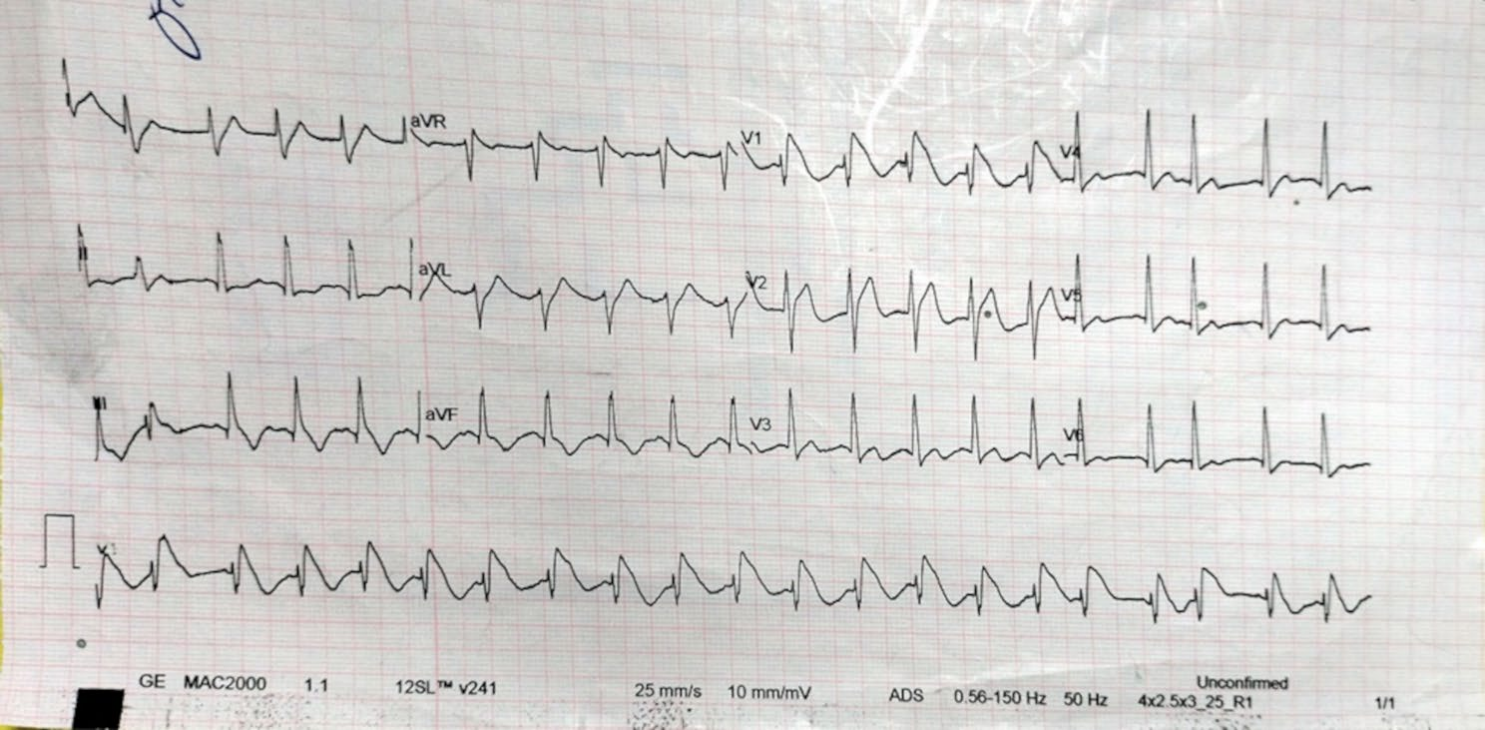
EKG of the patient

The patient was immediately put on I.V. normal saline, and an ECG was performed, which showed coved ST-segment elevation in V1 and V2 with T wave inversions in V1 to V3 (Brugada pattern) [Figure 1]. The venous blood gases showed severe metabolic and lactic acidosis [Table 1]. The laboratory blood work showed normal CBC and Na and K levels. The patient had a high troponin of 5275 ng/L, elevated CK of 304 U/L, elevated CK-MB of 81 U/L, elevated creatinine of 2.47 mg/dl, elevated ALT of 881 U/L, and elevated AST of 494 U/L. The patient also had a high serum amylase of 389 U/L, a PT of 34.1 s, a PTT of > 120 s, and an INR of 2.69. A pelvi-abdominal ultrasonography was performed, which showed mild to moderate free intraperitoneal fluid collection and mild to moderate bilateral pleural effusion. The ultrasonography also revealed ascites with a thickened, edematous gall bladder wall. The echocardiography was normal with an ejection fraction of 67% and mild tricuspid regurgitation.

**Table 1:** First VBG of the patient

pH	6.84
pCO2	37
Glu	240
Lac	> 15
HCO3	6.3

The patient was questioned multiple times alone and with his family it he had consumed any substance which he, again, denied. The patient’s full body was exposed, which showed no bite marks, scratch marks, or any rashes. The patient received 2000 mL of normal saline and was put on noradrenaline infusion (8 mg/500 mL D5W) when his blood pressure showed no improvement at first with 3 mL/hr, and by day 3, he reached 15 mL/hr. The patient was also given broad-spectrum antibiotics, anti-emetics, and proton pump inhibitors.

On day three, the patient admitted to one of the doctors that he had consumed an expired aluminum phosphide pellet, which he denied multiple times in fear of repercussions. Not long after this admission, the patient went into shock and tachypnea and was subsequently intubated. The patient then went into cardiac arrest and was moving from shockable to non-shockable rhythms multiple times and was eventually declared dead after 40 min of acute life support.

## Discussion

This case illustrates the rapid and devastating effects of AlP poisoning and underscores the difficulties in diagnosis when history from the patient is not forthcoming. The initial presentation of this patient with repeated vomiting, epigastric pain, and profound hypotension, in the absence of a clear toxicological history, presents a diagnostic challenge. His clinical deterioration, characterized by refractory hypotension, elevated cardiac enzymes, and metabolic derangements, was suggestive of severe systemic toxicity, though the source was initially unclear. This insidious course of disease progression, with the patient dying on the third day after presentation, also constituted a medical challenge due to the known rapid 24-hour mortality of AlP poisoning [3]. 

The ECG findings of coved ST-segment elevation in V1 and V2 with T wave inversions in V1-V3, while atypical, have been reported in cases of ALP poisoning and are often misinterpreted as signs of acute coronary syndrome [2, 4, 8, 11]. Elevated cardiac biomarkers in this case likely reflected direct myocardial damage due to phosphine gas rather than primary ischemia. Allam et al. [8] and Guru et al. [2] reported the occurrence of the Brugada pattern with AlP poisoning. However, they reported negative troponins in their case reports. AlP poisoning was also reported to be associated with STEMI with an ejection fraction of 15% in a 25-year-old man with no comorbidities [5]. Otherwise, published data on the association between cardiac arrhythmias and AlP poisoning was scarce and outdated [6, 7]. However, these outdated studies still reported the incidence of arrhythmias with AlP poisoning. For instance, Siwach et al. [6], have monitored the cardio-electrographic rhythms of 30 patients with AlP poisoning in 1998 and found that all patients had supraventricular and ventricular tachycardias, and as much as 40% of them had life-threatening ventricular tachycardia. However outdated, this warrants further investigation and might even warrant cardiac monitoring in patients with AlP poisoning.

Key laboratory findings in this patient, including elevated liver enzymes (ALT, AST), renal dysfunction (elevated creatinine), and coagulation abnormalities (prolonged PT, PTT, and INR), point to multi-organ involvement typical of ALP poisoning. Phosphine gas disrupts mitochondrial function, leading to widespread cellular damage, particularly affecting the heart, liver, and kidneys [12]. Additionally, the elevated serum amylase suggests possible pancreatic involvement, a less common but documented feature of ALP toxicity [13]. Moreover, the patient’s lab values showed significant hyperglycemia (240 mg/dl) before administration of D5W. A prospective cohort done by Mehrpour et al. reported that among 45 patients presenting with acute AlP poisoning, there was a statistically significant association between hyperglycemia (versus euglycemia) and mortality. However, there have also been reports of mortality associated with severe hypoglycemia [14, 15]. This underscores the importance of blood glucose monitoring and its importance in the prognostication of AlP poisoning.

The management of ALP poisoning is largely supportive, as there is no specific antidote. In this case, the patient was initially treated with aggressive fluid resuscitation and vasopressors to manage his profound hypotension. Unfortunately, his condition deteriorated despite these measures, which is a common outcome in ALP poisoning due to its high mortality rate. The administration of broad-spectrum antibiotics and proton pump inhibitors aimed to address possible secondary infections and prevent further gastrointestinal complications, though the primary pathology was driven by the toxic effects of phosphine gas. However, there has been a rising number of experimental clinical studies suggesting the use of magnesium sulfate, melatonin, N-acetylcystein, glutathione, sodium selenite, and vitamin C and E, among others, to reduce the hazardous oxidative properties of AlP [16–18]. Moreover, there have been reports of patients benefiting from extracorporeal membrane oxygenation (ECMO) in cases of acute AlP poisoning [19–21]. However, these treatments are still experimental and mostly inapplicable in the low-resource setting where AlP poisoning is prevalent.

This case highlights the importance of early recognition and intervention in suspected cases of ALP poisoning. However, the diagnostic process was delayed by the patient’s reluctance to admit to ingesting ALP, which is a common scenario in toxicology cases, particularly when patients fear legal or familial repercussions. The reluctance to disclose toxic ingestion emphasizes the need for a thorough and nonjudgmental history-taking approach, as well as a high index of suspicion in cases of unexplained shock, metabolic acidosis, and organ failure. Moreover, the diagnosis of this case was hindered but the insidious progression of the case, which in hindsight, could have been attributed to the expired AlP pellet. Despite the best efforts, the prognosis for patients with ALP poisoning remains poor, particularly in cases with delayed presentation or lack of early intervention. The patient’s progression to cardiac arrest despite aggressive treatment demonstrates the difficulty of reversing the toxic effects once multi-organ dysfunction has set in. Phosphine-induced cardiotoxicity and the refractory shock seen in this case are particularly resistant to treatment, often leading to fatal outcomes. Clinicians, especially in rural or low-resource settings, should maintain a high suspicion for AlP poisoning in patients with unexplained shock, metabolic acidosis, or Brugada-like ECG changes. Early supportive care, cardiac and glucose monitoring, and empathetic, nonjudgmental history-taking are essential to improve diagnosis and outcomes.

In conclusion, ALP poisoning remains a critical challenge in toxicology, particularly in areas where access to the substance is widespread. Early diagnosis and supportive care are crucial, but outcomes are often grim, as seen in this case. This case underscores the need for heightened awareness of AlP poisoning, particularly in patients presenting with unexplained gastrointestinal symptoms and shock, and highlights the importance of timely disclosure of toxic exposures to optimize management and improve prognosis, and the importance of glucose monitoring in prognostication of AlP poisoning patients. Furthermore, this case underscores the importance of increasing the medical suspicion regarding toxicological etiology, especially AlP poisoning, among acutely ill patients living in rural areas presenting with incomplete or vague history. Finally, the presentation of Brugada ECG in patients with the aforementioned presentation and vague history should also hint to AlP poisoning when no other medical diagnoses fit.

## Electronic supplementary material

Below is the link to the electronic supplementary material.

Supplementary Material 1

Supplementary Material 2

## Data Availability

No datasets were generated or analysed during the current study.
