# Supplementary material for: Aluminum phosphide poisoning with Brugada ECG: a case report highlighting diagnostic challenges arising from patient nondisclosure
Source: Int J Emerg Med. 2025 May 12;18:96. doi: 10.1186/s12245-025-00899-z (PMC12067657; doi:10.1186/s12245-025-00899-z)
Supplement: Supplementary file 1 — Supplementary Material 1 [file 12245_2025_899_MOESM1_ESM.docx]

| pH | 6.84 |
| --- | --- |
| pCO2 | 37 |
| Glu | 240 |
| Lac | >15 |
| HCO3 | 6.3 |
