# Supplementary figures and images for: Aluminum phosphide poisoning with Brugada ECG: a case report highlighting diagnostic challenges arising from patient nondisclosure
Source: Int J Emerg Med. 2025 May 12;18:96. doi: 10.1186/s12245-025-00899-z (PMC12067657; doi:10.1186/s12245-025-00899-z)

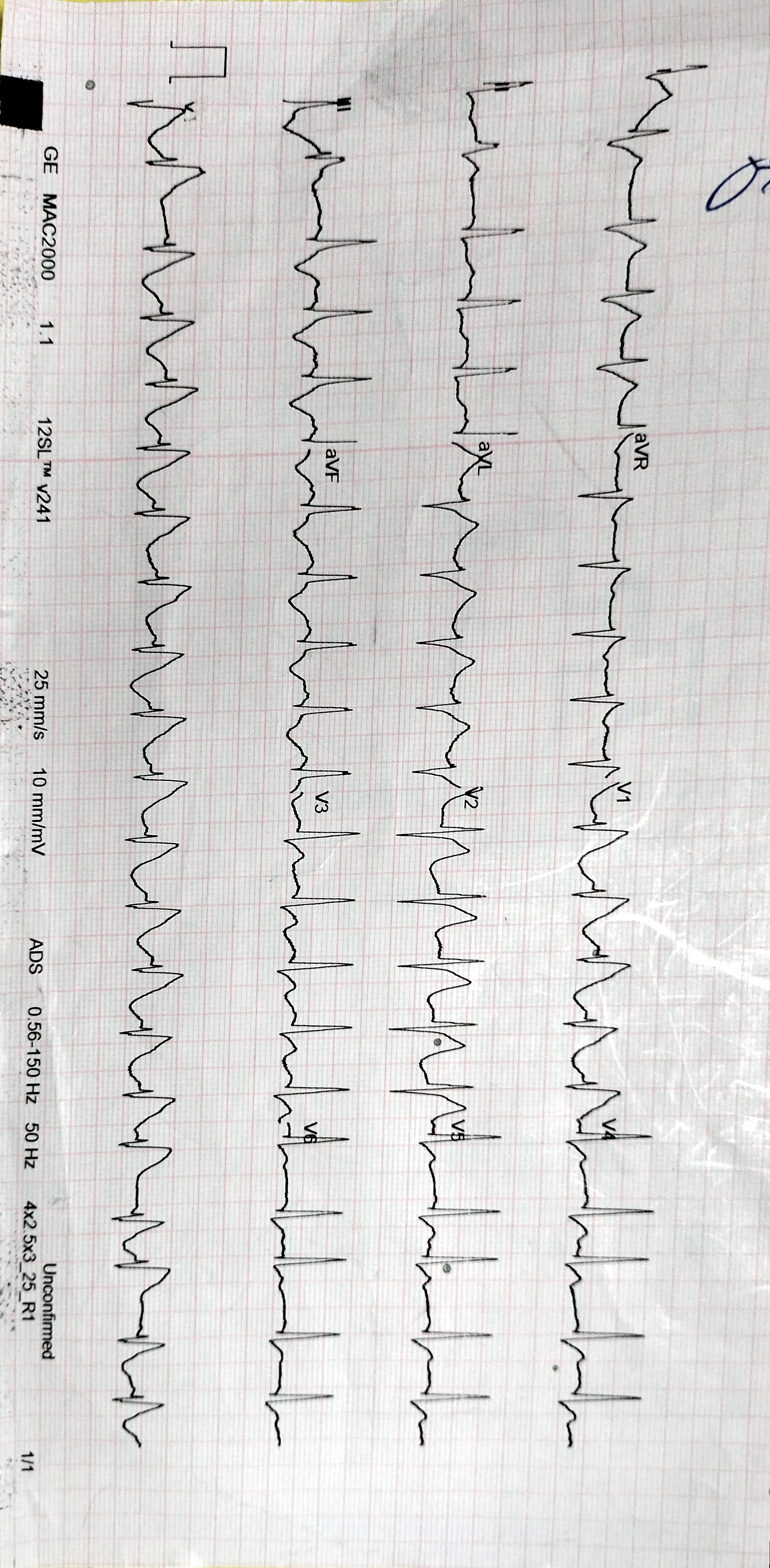

Supplement: Supplementary file 2 — Supplementary Material 2 [file 12245_2025_899_MOESM2_ESM.jpg]
